# Geographically Weighted Random Forest Considering Spatial Heterogeneity for Landslide Susceptibility Assessment: A Case Study in Yingjiang County

**DOI:** 10.3390/s26041142

**Published:** 2026-02-10

**Authors:** Weiheng Qian, Mengyao Shi, Cheng Huang, Huan Li, Junjie Huang

**Affiliations:** 1 Faculty of Land Resources Engineering, Kunming University of Science and Technology, Kunming 650093, China; qianweiheng@stu.kust.edu.cn (W.Q.); lihuan@stu.kust.edu.cn (H.L.); 20242201097@stu.kust.edu.cn (J.H.); 2 Yunnan Key Laboratory of Intelligent Monitoring and Spatiotemporal Big Data Governance of Natural Resources, Kunming University of Science and Technology, Kunming 650093, China; 3 Yunnan Institute of Geo-Environment Monitoring, Kunming 650216, China; hch2377@stu.kust.edu.cn

**Keywords:** landslide susceptibility assessment, geographically weighted random forest, spatial heterogeneity, Yingjiang

## Abstract

Landslide susceptibility mapping (LSM) is widely used for identifying potential landslide-prone areas. However, many existing approaches rely on global models that assume spatial stationarity, which limits their ability to capture spatially heterogeneous relationships in complex mountainous regions. To address this issue, this study improved landslide susceptibility evaluation by accounting for spatial heterogeneity using a Geographically Weighted Random Forest (GWRF) model. By allowing the influence of conditioning factors to vary spatially, the proposed method provides a more adaptive representation of landslide susceptibility compared to conventional global models. The GWRF-based evaluation results were compared with those obtained from Random Forest (RF) and XGBoost models to examine relative performance. The study was conducted in Yingjiang County, a landslide-prone mountainous area, using multiple landslide conditioning factors, including topographic and anthropogenic variables such as slope and distance to roads. Landslide susceptibility maps were generated, and the evaluation results were supported by InSAR-derived deformation data, field investigations, and UAV observations. The results indicate that the GWRF model achieved superior overall susceptibility evaluation performance compared to the RF and XGBoost models, with an AUC value of 0.922. Furthermore, compared to global models, the GWRF model revealed more detailed spatial patterns of landslide susceptibility, particularly in high-susceptibility zones. Areas classified as highly susceptible by the GWRF model also demonstrated greater consistency with observed deformation features. These findings highlight the importance of considering spatial heterogeneity in landslide susceptibility evaluation and demonstrate that the proposed GWRF approach is applicable for regional-scale susceptibility assessment in complex mountainous environments.

## 1. Introduction

Landslides are widespread hazards in mountainous regions, often triggered by complex interactions between geological conditions and external environmental factors, posing significant threats to human safety and infrastructure [1,2,3]. Identifying potential landslide zones and creating accurate Landslide Susceptibility Maps (LSM) are fundamental prerequisites for prevention and mitigation. Currently, LSM primarily relies on data-driven methods, particularly machine learning algorithms such as Random Forest (RF), Support Vector Machines (SVM), and XGBoost. These models have gained prominence due to their strong capability to handle non-linear relationships and high predictive accuracy [4,5].

However, a critical limitation of these traditional machine learning models is their assumption of uniform spatial relationships. They typically treat the relationship between landslide occurrence and conditioning factors (e.g., slope, lithology, rainfall) as constant across the entire study area. In reality, the inducing mechanisms of landslides often exhibit significant spatial heterogeneity due to variations in local geological environments. For instance, rainfall may be the dominant triggering factor in a valley region, while tectonic activity (e.g., distance to faults) may control landslide distribution at higher elevations [6]. Global models (like RF and XGBoost) homogenize these local variations, leading to smoothed prediction results that may underestimate risks in locally complex areas or fail to capture specific deformation patterns [7].

To address spatial heterogeneity, local modeling techniques have been developed. Geographically Weighted Regression (GWR) is a classic approach that allows regression coefficients to vary spatially [8]. However, traditional GWR is a linear model and struggles to capture the complex, non-linear interactions inherent in landslide formation. Consequently, the Geographically Weighted Random Forest (GWRF) model was proposed by integrating the spatial weighting mechanism of GWR with the powerful non-linear fitting ability of RF [9,10]. Despite its potential, the application of GWRF in landslide susceptibility assessment—specifically its comparative performance against state-of-the-art global models like XGBoost—requires further empirical validation.

In recent years, although a growing number of studies have applied advanced machine learning and ensemble models to LSM with notable improvements in accuracy, most still rely on global modeling strategies that assume spatially stationary relationships. Limited attention has been paid to local geological variability and the spatial heterogeneity of landslide-controlling mechanisms. While several studies have begun to explore geographically weighted or locally adaptive models, their application remains relatively limited, and systematic comparisons with state-of-the-art global models are scarce. In particular, the potential advantages of GWRF in capturing spatial heterogeneity under complex geological settings have not been sufficiently examined through comprehensive case studies.

Moreover, existing landslide susceptibility studies predominantly focus on static model validation using historical landslide inventories and statistical accuracy metrics. The integration of dynamic deformation observations into susceptibility evaluation is still at an early stage, and few studies have quantitatively linked InSAR-derived ground deformation with susceptibility classes to assess model rationality. As a result, it remains unclear whether models that explicitly consider spatial heterogeneity, such as GWRF, are more effective in identifying actively deforming or potentially unstable slopes compared to conventional global models.

These gaps highlight the need for an integrated framework that simultaneously evaluates the role of spatial heterogeneity in susceptibility modeling and incorporates independent deformation observations for model validation. Furthermore, while traditional LSM focuses on “static” probability based on historical inventories, Interferometric Synthetic Aperture Radar (InSAR) technology provides a powerful tool to verify whether identified high-susceptibility zones are actually active by detecting millimeter-level surface deformation over large areas [11,12,13]. Integrating the static spatial predictions of GWRF with the dynamic monitoring data of InSAR offers a promising pathway for more reliable landslide assessment.

Therefore, this study takes Yingjiang County, a region with complex topography and frequent landslides, as the study area to propose an integrated assessment framework. The primary objectives are: (1) to calculate the line-of-sight velocity map using Sentinel-1 data; (2) to construct and compare GWRF, RF, and XGBoost models to evaluate the necessity of considering spatial heterogeneity in LSM; and (3) to validate the model results using InSAR deformation data, specifically analyzing whether GWRF can better identify active landslide zones that global models might miss. This study aims to provide new insights into interpreting the spatial variability of landslide mechanisms and improving the precision of regional hazard prevention.

The remainder of this paper is organized as follows: Section 2 systematically introduces the study area, data sources, and the preparation of Landslide Conditioning Factors (LCFs). Section 3 elaborates on the theoretical basis of the methodology, detailing the mathematical formulation and construction process of the GWRF model. Section 4 presents the multicollinearity test results for LCFs, compares the Landslide Susceptibility Maps (LSMs) generated by the GWRF, RF, and XGBoost models, and evaluates their predictive accuracy. Section 5 discusses the advantages of the GWRF model, analyzes the spatial heterogeneity and feature importance derived from the models, validates the results through field investigations, and explores the implications for disaster prevention and control. Finally, Section 6 concludes the paper.

## 2. Study Area and Data Sources

### 2.1. Study Area

**Figure 1:**
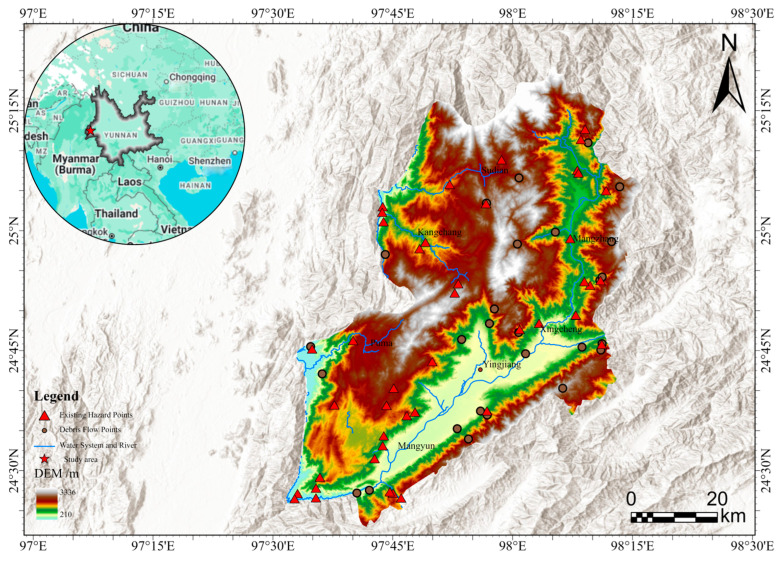
Location and topographic map of the study area. The main map shows elevation distribution with major rivers (blue lines) and the spatial distribution of hazards. The top-left inset map shows the approximate geographical location of the study area in Yunnan Province.

The geographical area under scrutiny in this study is Yingjiang County, which falls under the jurisdiction of Dehong Dai and Jingpo Autonomous Prefecture in Yunnan Province, China. The area’s geographical location is specified by the following coordinates: 97°31′40″ E to 98°15′00″ E and 24°24′16″ N to 25°20′10″ N, as illustrated in Figure 1. At the southwestern end of the southern extension of the Gaoligong Mountains, the terrain experiences a gradual descent from northeast to southwest. The region is distinguished by a distinctive topography, characterised by an alternating pattern of mountains and valleys. These mountains and valleys are distributed unevenly across the region, with higher elevations concentrated in the north-eastern part and lower elevations concentrated in the south-western part. This topographic pattern is indicative of significant variations in the region’s landscape, with a general trend of higher elevations in the north-eastern part and lower elevations in the south-western part. Yingjiang County has an area of 4429 square kilometres, which is approximately 38.4% of the total land area of the prefecture. The county is traversed by 43 major rivers, which are divided into four primary river systems: the Dayingjiang River system, the Jieyang River system, the Mengga River system, and the Longchuan River system. The county is subject to a South Asian subtropical monsoon climate. Due to the varied geomorphological compositions and marked elevation differences, there are substantial climatic variations across different regions, ranging from tropical to subtropical and temperate zones. The phenomenon of vertical climatic zoning is characterised by the presence of multiple climate types within the same administrative unit. This results in the formation of a distinctive “three-dimensional climate” combination. From an administrative perspective, Yingjiang County comprises a total of 15 administrative subdivisions, namely eight towns and seven townships. As demonstrated in the results of the Seventh National Population Census, the county’s resident population was 292,508 as of 00:00 on 1 November 2020.

### 2.2. Field Photographs of Landslides

**Figure 2:**
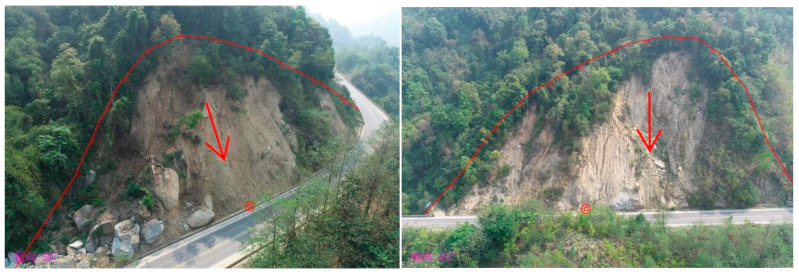
Field photograph of a road-cut–induced landslide in Nongzhang. Red arrows indicate the sliding direction. The red line indicates the landslide perimeter, and the red circles represent the coordinate points generated by the software.

A field investigation was conducted in Nongzhang Township, Yingjiang County, where landslides induced by road cutting are a common occurrence. As demonstrated in Figure 2, the case under consideration is that of a representative incident instigated by highway excavation. The occurrence of slope failure, as evidenced by the presence of exposed soil layers and displaced material, underscores the substantial impact of engineering activities on slope stability within this geographical area.

### 2.3. Data Sources

The formation of landslides is governed by the coupled effects of multiple agents, including topography, geomorphology, geological structures, and hydro-meteorological conditions. The influences of these elements exhibit pronounced spatial heterogeneity, meaning that their contributions to landslide occurrence vary significantly across space. Consequently, the GWRF model was constructed using a selection of landslide conditioning factors based on the environmental characteristics of the study area. In order to capture local variations, it was necessary to prioritise key factors such as slope and rainfall. As delineated in Table 1, exhaustive details pertaining to the data sources are furnished. The subsequent Section 2.5 is dedicated to the exposition of the data preprocessing procedures.

### 2.4. SAR Data

**Figure 3:**
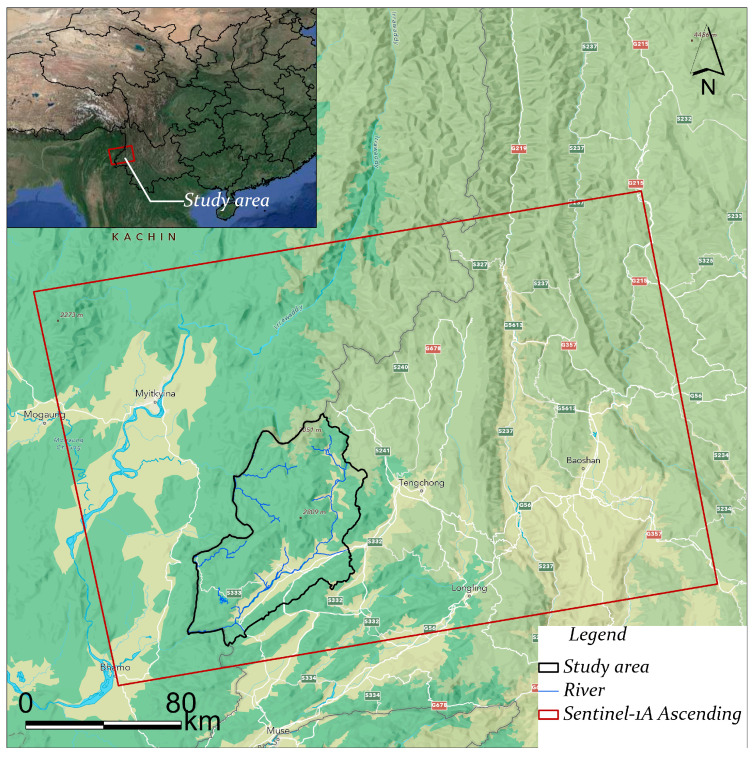
Spatial coverage of Sentinel-1A SAR data and major river distribution in the study area. The upper-left inset illustrates the overall SAR image coverage, with the red polygons representing different SAR acquisition frames, while the blue lines indicate the main river network.

In order to accurately capture the latest surface deformation characteristics and meet the precision requirements of time series interferometric synthetic aperture radar (InSAR) analysis, this study primarily utilizes SAR data acquired from the Sentinel-1A satellite [14], which is part of the European Space Agency’s Copernicus Program (GMES). The Sentinel-1 constellation currently consists of two satellites (Sentinel-1A and Sentinel-1B), both of which operate in the C-band (wavelength: 5.6 cm) and are capable of multipolarisation synthetic aperture radar imaging. The satellites in question are capable of providing consistent, high-resolution Earth observation data under all-weather and all-day conditions. This encompasses day/night and various weather scenarios. In the present study, a total of 33 ascending-pass SAR scenes in interferometric wide (IW) mode and VV polarization were selected, covering the period from December 2024 to December 2025, as outlined in Table 2. The spatial distribution of the SAR data and the designated study area are delineated in Figure 3.

### 2.5. Landslide Conditioning Factors

In light of the absence of a universally accepted methodology for the selection of landslide conditioning factors (LCFs), this paper employs a comprehensive literature review [2,15] and a thorough analysis of the geological and environmental characteristics of the study area to identify 10 LCFs. These factors are derived from various data sources and are categorised into topographic, land-related, geological, and water-related factors. The selected LCFs comprise elevation, slope, aspect, curvature, FVC, profile curvature, topographic wetness index (TWI), distance to roads, distance to rivers, distance to faults, and annual precipitation. Given the scale of the study area, the spatial distribution of landslides, and the computational complexity of the model, all thematic maps are converted to a raster format with a resolution of 30 m × 30 m in GIS. Consistent with this evaluation unit, the landslide inventory employed in this study primarily captures landslide events discernible at this scale, where the minimum mappable size is implicitly constrained to approximately 900 m^2^

**Figure 4:**
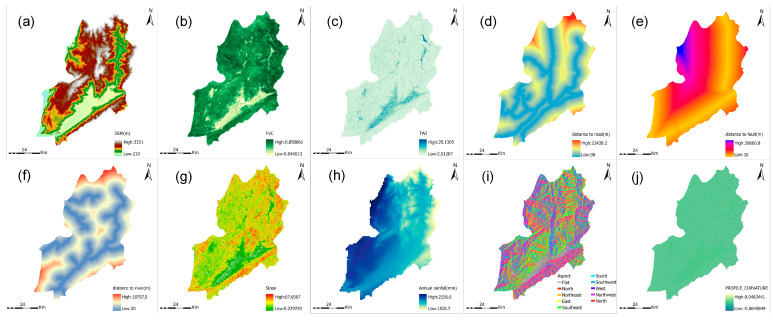
Thematic Map of Landslide Influencing Factors: (**a**) DEM; (**b**) FVC; (**c**) TWI; (**d**) Distance to road; (**e**) Distance to fault; (**f**) Distance to river; (**g**) Slope; (**h**) Annual rainfall; (**i**) Aspect; (**j**) profile curvature.

A total of ten conditioning factors were selected for the landslide susceptibility assessment and subsequently processed. The following parameters were analysed: DEM, FVC, TWI, distance to road, distance to fault, distance to river, slope, annual rainfall, aspect, and profile curvature (Figure 4). The number of conditioning factors was determined by balancing geomorphic relevance, data availability, and model robustness. It has been demonstrated by earlier research that the capacity to predict susceptibility does not necessarily increase to a significant extent by including a greater number of conditioning factors, provided that a reasonable and representative set is employed. Instead, the relevance and complementarity of the selected predictors are more important than their sheer quantity. Therefore, the ten factors that were adopted were selected to cover key topographic, hydrological, anthropogenic, tectonic, and climatic controls on landslides, while avoiding unnecessary redundancy and excessive computational complexity [16].

The DEM data, which had a spatial resolution of 30 m, was obtained from the original dataset and utilised directly. The FVC was calculated using the Google Earth Engine (GEE) platform, based on remote sensing imagery and NDVI transformation. The annual rainfall data were derived from the National Tibetan Plateau Scientific Data Center [17].

A range of topographic factors was computed in ArcGIS Pro 3.4, including slope, aspect, TWI, and profile curvature. These computations were performed using the Spatial Analyst toolbox, with the Digital Elevation Model (DEM) data as the underlying source.

Distance factors were generated by calculating the Euclidean distance from each pixel to the nearest road, fault line, and river, respectively, using ArcGIS. The distance to road, distance to fault, and distance to river were thus calculated. All factors were resampled to a uniform 30 m resolution, normalized to a range of 0–1, and imported into the machine learning models for susceptibility calculation.

The present study employed two methods to screen suitable landslide factors. Initially, the Pearson Correlation Coefficient (PCC) was determined in order to assess the correlation between factors. Factors demonstrating strong correlations with other factors (|r| > 0.7) were considered redundant. Subsequently, the variance inflation factor (VIF) was employed to evaluate the multicollinearity among LCFs. A VIF greater than 10 was considered an indicator of significant multicollinearity and was therefore excluded [18].

In order to circumvent the conceptual ambiguity between landslide susceptibility and event-based hazard triggering, it is imperative to elucidate that the rainfall-related factors employed in this study do not denote short-term or event-specific triggering conditions [19]. Instead, these variables are intended to characterize the long-term hydro-climatic background of the study area [20].

The long-term rainfall conditions exert a significant influence on slope stability through a series of cumulative effects, including the regulation of soil moisture, the intensity of weathering processes, and hydrological processes. These factors collectively contribute to the spatial predisposition of landslide occurrence. Consequently, rainfall is incorporated in this study as a regional-scale environmental conditioning factor reflecting persistent hydrological forcing, rather than as an external trigger for individual landslide events.

## 3. Methods

**Figure 5:**
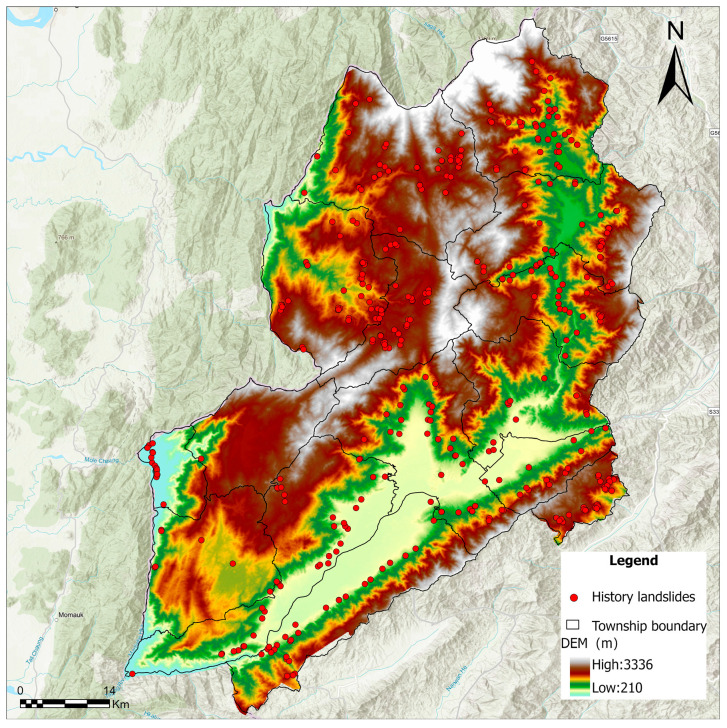
Distribution of historical landslide occurrences overlaid on the Digital Elevation Model (DEM).

It is imperative to acknowledge that a reliable landslide inventory constitutes the fundamental prerequisite for susceptibility modelling. In the course of this study, a total of 445 historical landslide occurrences were compiled for the purpose of constructing the inventory database. The spatial distribution of these landslides is illustrated in Figure 5, in which the landslide points are overlaid on the Digital Elevation Model (DEM) to visualise their relationship with the topographic terrain.

The construction of a robust machine learning model is contingent upon the quality of non-landslide samples (negative samples); therefore, a rigorous selection strategy was employed to generate a balanced dataset. Addressing the issue of class imbalance common in landslide modeling [21], a 1:1 ratio of landslide to non-landslide samples was adopted to prevent model bias towards the majority class. To select these negative samples, a 500-m buffer zone was established around each identified landslide. Given the 30-m spatial resolution, this buffer creates a separation of approximately 17 pixels between positive and negative samples. This physical gap is deemed sufficient to minimize spatial autocorrelation and reduce the risk of including potentially unstable adjacent slopes in the negative dataset, a sampling bias known to degrade model quality [14]. From the stable areas outside these buffer zones, 445 non-landslide samples were randomly selected. While we acknowledge that the specific buffer distance and sampling ratio may influence the model’s transferability to regions with different topographic frequencies, this procedure ensures that the current training dataset is balanced, representative, and spatially independent.

Standard RF is a global regression/classification method. The fundamental assumption underpinning this method is that the relationship between landslide occurrence and its conditioning factors is spatially stationary. However, it is important to note that geological environments frequently exhibit significant spatial heterogeneity. In order to capture this non-stationarity, the Geographically Weighted Regression (GWR) framework is integrated with the local weighting mechanism of GWR within the RF framework. The specific content of this phenomenon will be elaborated in the following sections. The landslide susceptibility index (LSI) was predicted through the following methodology. Firstly, landslide samples were assigned the label “1”, whereas non-landslide samples were assigned the label “0”. The landslide and non-landslide data sets were randomly divided into two subsets: 70% of the data was used for model training, and the remaining 30% was allocated for model validation [22]. Utilize the raster calculator of GIS to perform normalization and pixel alignment on LCFs, and conduct VIF testing and Pearson correlation coefficient verification [23].

**Figure 6:**
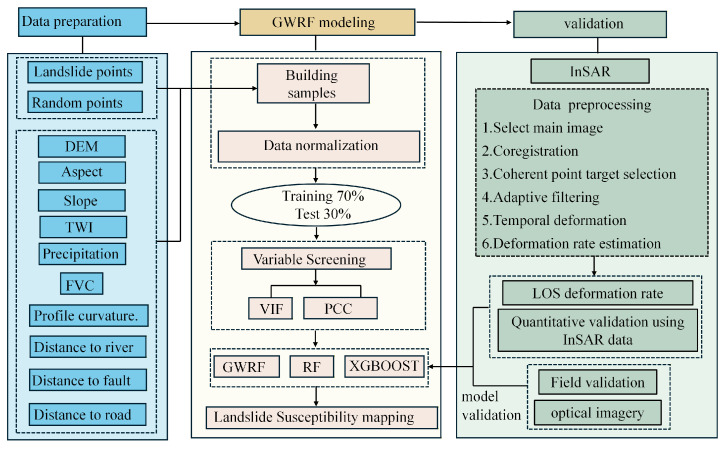
Landslide susceptibility evaluation flowchart showing the integrated approach of InSAR and machine learning.

This study conducts landslide susceptibility modeling using GWRF, RF, and XGBoost, and subsequently employs time-series InSAR techniques to derive the line-of-sight (LOS) deformation rate for validating the high-susceptibility zones identified by the three models (Figure 6). Furthermore, spatial heterogeneity characteristic maps were generated by differencing the GWRF results with those of the RF and XGBoost models, enabling a focused comparison of the spatial heterogeneity patterns of selected landslide conditioning factors (LCFs).

### 3.1. GWR

The GWR model is an extension of multiple linear regression in which regression coefficients vary spatially to account for geographic heterogeneity. By explicitly incorporating geographic location into the regression framework, GWR enables local relationships between explanatory variables and the response variable to be modeled, thereby effectively capturing spatial nonstationarity [8,24]. The model can be expressed as follows:

(1) $$y_{i}=\beta_{0}\left(u_{i},v_{i}\right)+\overset{p}{\underset{k=1}{\sum }}\beta_{k}\left(u_{i},v_{i}\right)x_{ik}+\varepsilon_{i}$$

where $y_{i}$ is the response at location i with coordinates $\left(u_{i},v_{i}\right)$; $x_{ik}$ is the k-th predictor; $\beta_{0}(u_{i},v_{i})$ and $\beta_{k}(u_{i},v_{i})$ are spatially varying intercept and coefficients; p is the number of predictors; and $\varepsilon_{i}$ is the error term.

### 3.2. Design of GWRF

Landslide susceptibility is inherently spatially non-stationary, meaning the relationship between conditioning factors and slope stability varies across the landscape [25]. Traditional global models (e.g., standard Random Forest) often fail to capture these local variations. To address this, we implemented a Geographically Weighted Random Forest (GWRF), which extends the ensemble learning capabilities of Random Forest into the spatial domain [9].

Unlike hybrid approaches that merely use GWR coefficients as input features, our GWRF implementation directly embeds spatial heterogeneity into the training process via local sample weighting. Specifically, the model consists of an ensemble of decision trees, where each tree acts as a “local expert” centered on a randomly selected spatial anchor. The contribution of training samples to each tree is weighted by a spatial kernel function [24].

To account for the uneven distribution of landslide samples, an adaptive bandwidth strategy was employed for the kernel function. In contrast to a fixed bandwidth, this adaptive scheme dynamically adjusts the spatial extent of the kernel based on local sample density, ensuring that each local estimator has access to a sufficient number of observations [2]. This strategy enhances the robustness of parameter estimation in sparsely sampled regions while preserving fine-scale details in data-dense areas. By aggregating the predictions of these locally weighted trees, the GWRF framework effectively models the complex, spatially varying nonlinear interactions among environmental factors.

Landslide susceptibility exhibits significant spatial non-stationarity where the causal relationships between environmental conditioning factors and slope instability vary across the geographical landscape. To address this spatial heterogeneity effectively, this study implements a Geographically Weighted Random Forest model. This approach extends the ensemble learning paradigm by constructing a collection of locally sensitized decision trees, thereby integrating spatial dependence directly into the machine learning framework without relying on a two-step feature extraction process.

**Figure 7:**
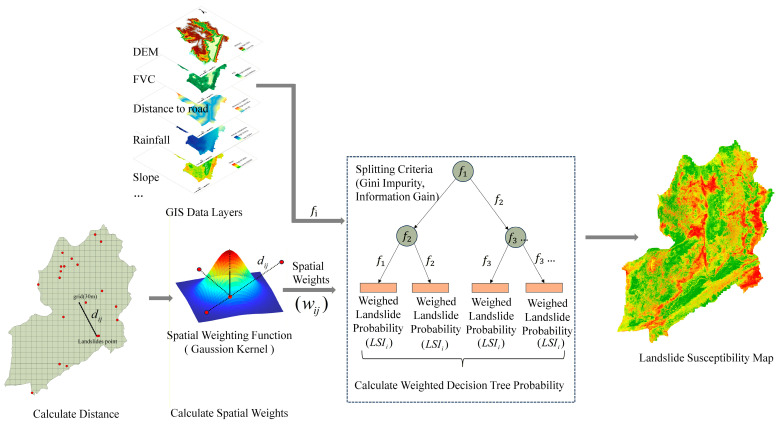
Schematic of the Geographically Weighted Random Forest (GWRF) workflow for landslide susceptibility mapping, including input data, spatial weight calculation, weighted model training, and susceptibility output. The red points represent a subset of landslide points.

To address this spatial heterogeneity effectively, this study implements a Geographically Weighted Random Forest model using Python 3.9 with the scikit-learn and NumPy libraries. As illustrated in Figure 7.

#### 3.2.1. Spatial Weighting Strategy and Kernel Function

The fundamental mechanism distinguishing the GWRF from a global Random Forest is the assignment of spatial weights to training samples based on their proximity to specific reference locations. For the construction of each base estimator within the ensemble, a coordinate is randomly sampled from the training dataset to serve as a spatial anchor point, denoted as $u_{k}$ for the k-th tree.

The influence of each training sample on the development of a specific tree is determined by a spatial kernel function. A Gaussian kernel is employed to compute a weight $w_{i,k}$ for every training observation i located at coordinate $x_{i}$. This weight reflects the Euclidean distance between the observation and the anchor point of the current tree, ensuring that the influence of a sample decays smoothly as the distance increases. The weighting formula is defined as:

(2) $$w_{i,k}=\exp\left(-\frac{1}{2}{\left(\frac{∥x_{i}-u_{k}∥}{h}\right)}^{2}\right)$$

In this equation, $∥x_{i}-u_{k}∥$ represents the Euclidean distance between the training sample and the anchor point, while h signifies the bandwidth parameter, which controls the rate of spatial decay and determines the effective range of local influence for each estimator.

#### 3.2.2. Locally Weighted Tree Induction

Once the spatial weights are established for a given tree, they are integrated directly into the induction algorithm of the Decision Tree classifier. Unlike standard implementations where samples are treated uniformly, the GWRF utilizes these weights to modify the splitting criterion at each node. Specifically, the Gini Impurity calculation is adjusted to ensure that samples possessing higher spatial weights due to their proximity to the anchor point contribute more significantly to the evaluation of split quality.

This process ensures that each tree in the forest functions as a local expert specialized in modeling the landslide mechanisms pertinent to the vicinity of its anchor point. To preserve the variance-reduction benefits of the ensemble method, the randomized subspace strategy is retained, where a random subset of features is selected as candidates for each node split. This combination of spatial weighting and random feature selection allows the model to capture local spatial dependencies while mitigating the risk of overfitting associated with individual local models.

#### 3.2.3. Probability Surface Generation

The final landslide susceptibility assessment is derived through an ensemble aggregation process. For any unobserved location $x_{new}$ in the study area, the input feature vector is passed through all T trees in the forest. Each tree $f_{k}$ produces a class probability estimate $P_{k}(y=1|x_{new})$ based on the learned local patterns. The definitive Landslide Susceptibility Index is computed as the arithmetic mean of the probabilities predicted by all constituent estimators:

(3) $$LSI(x_{new})=\frac{1}{T}\overset{T}{\underset{k=1}{\sum }}P_{k}(y=1|x_{new})$$

This soft voting mechanism effectively smooths the predictions across the study area, resulting in a continuous probability surface ranging from 0 to 1, where higher values indicate a greater likelihood of landslide occurrence.

## 4. Results

### 4.1. Factor Selection Results

The GWRF model combines spatial weighting and ensemble learning; as such, a two-step collinearity diagnosis was implemented. VIF analysis is employed chiefly to address the sensitivity of the geographically weighted component to multicollinearity [18]; Pearson correlation coefficients are subsequently plotted to identify pair-wise correlations and to reduce feature redundancy. This may lead to biases in variable importance assessment within the RF module [26].

**Figure 8:**
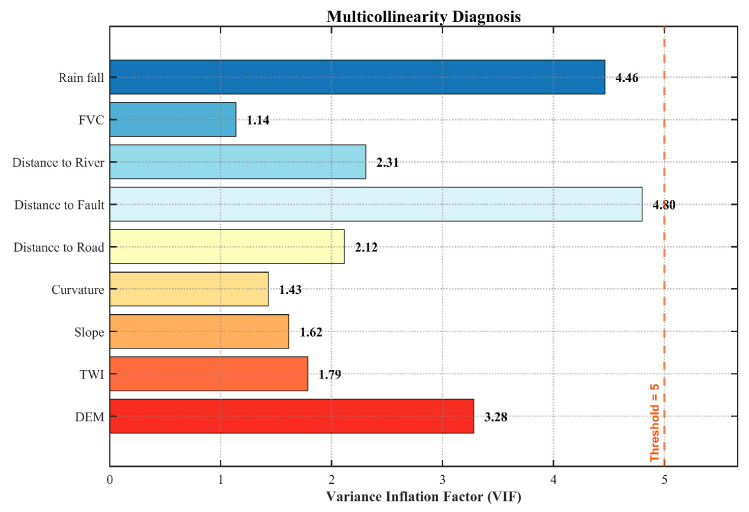
Multicollinearity analysis of LCFs.

Based on the strict criterion of VIF ≤ 5, the Aspect factor was identified as redundant and subsequently excluded from the analysis. As illustrated in Figure 8, the VIF values for all retained conditioning factors strictly adhere to this limit, confirming that multicollinearity is effectively minimized. Consequently, the selected dataset is deemed statistically independent, providing a stable and reliable foundation for the subsequent GWRF modeling.

These findings suggest that there is a weak correlation among the independent variables and that multicollinearity is not a significant issue. It is therefore concluded that the selected variables are deemed to be independent, thus providing effective support for subsequent modelling and ensuring the stability and reliability of the model.

**Figure 9:**
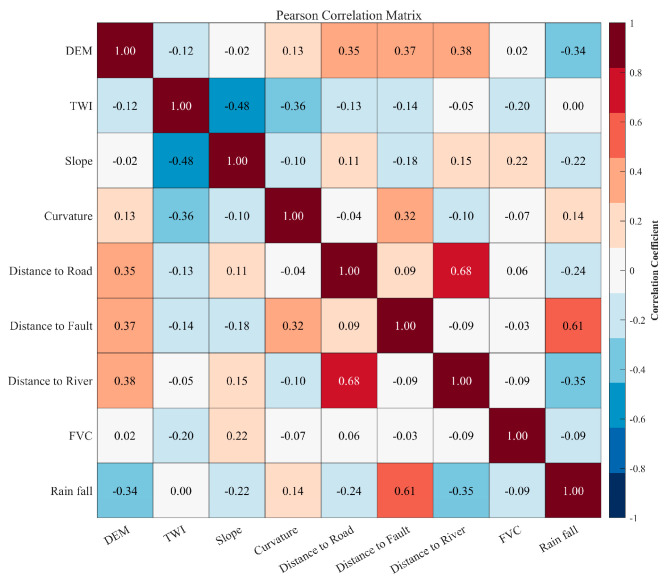
Pearson correlation coefficient matrix between LCFs.

The subsequent stage of the process involves the utilisation of the variable correlation matrix to reflect the Pearson correlation coefficients between multiple variables, thereby indicating their linear correlations. As illustrated in Figure 9 below, the correlation coefficients between the nine variables are demonstrated.

It is important to acknowledge that while global VIF screening minimizes overall redundancy, residual local multicollinearity may persist in specific sub-regions due to the spatial heterogeneity of environmental variables. Unlike traditional Geographically Weighted Regression, where such local collinearity often leads to unstable coefficient estimates and sign reversals, the GWRF model employed in this study demonstrates superior robustness. This resilience stems from the decision tree-based learner, which utilizes a random subspace method that selects a random subset of features at each node split. This mechanism effectively decorrelates the trees and ensures that the model can utilize information from correlated features without suffering from the estimation variance that typically affects linear spatial regression models, thereby preserving the reliability of local landslide susceptibility assessments.

### 4.2. Relationship Between Landslides and Conditioning Factors

**Figure 10:**
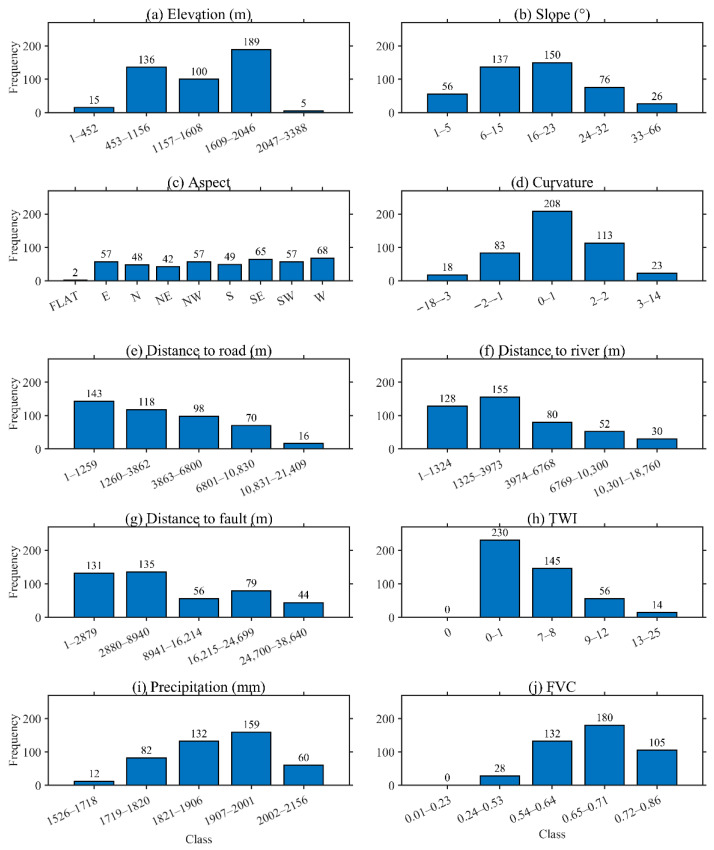
Relationship between landslides and LCFs.

The present study sought to analyse the relationship between landslide occurrence and LCFs in the designated study area. The relationship between the LCFs and landslide occurrence was analysed in the study area, as demonstrated in Figure 10. Landslides are predominantly concentrated at mid-elevations, with the highest frequency occurring in the altitude range of 1609–2046 m, whereas very high elevations exhibit a markedly lower incidence of landslides. With respect to slope, landslides predominantly occur on moderate slopes, peaking at 16–23° and 6–15°, while very steep slopes exhibit lower frequencies, suggesting that extremely steep terrain contributes less to landslide accumulation in the inventory.

The aspect distribution does not demonstrate a clear directional dominance; however, comparatively elevated frequencies are observed on west-facing and southeast-facing slopes. The analysis of curvature indicates that excessively high and low curvature values do not significantly promote landslide occurrence. Conversely, landslides are predominantly observed in near-zero curvature classes, suggesting that relatively smooth geomorphic conditions are more susceptible to slope failure in the study area.

A consistent proximity pattern is observed for faults, rivers, and roads. The proximity of landslides to these factors is directly correlated with the probability of landslide occurrence, with a general decrease in frequency observed as distance increases. Furthermore, landslides are known to be concentrated under low TWI conditions and moderate-to-high FVC levels. Precipitation exhibits a discernible concentration pattern, with landslides manifesting most frequently under relatively higher rainfall classes. This observation suggests that wetter climatic conditions may potentially promote slope instability in the study area.

### 4.3. InSAR Deformation Rate

Strict Masking Strategy for Geometric Distortions: To ensure the reliability of the InSAR data, we implemented a rigorous quality-control strategy within the SBAS-InSAR workflow. Specifically, we:

- Applied a coherence threshold (coherence < 0.3) to exclude low-quality pixels.
- Explicitly masked layover and shadow areas based on the Local Incidence Angle (LIA) and slope aspect derived from the DEM.

SBAS-InSAR Processing Workflow:

The SBAS-InSAR time-series analysis was implemented using the GAMMA 2023 software package with its Interferometric Point Target Analysis (IPTA) module [27]. The detailed processing workflow is as follows:

(1) Baseline Configuration and Image Selection

**Figure 11:**
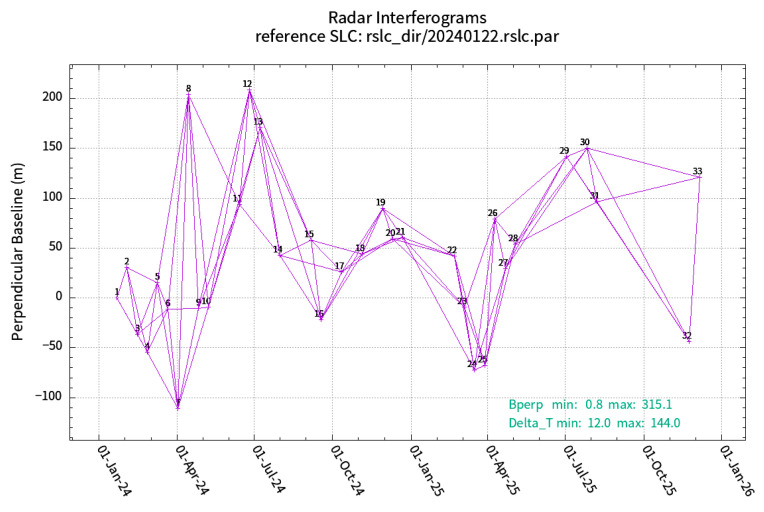
Spatio-temporal baseline network of SBAS-InSAR processing. The perpendicular baseline threshold is set to 315 m, and the temporal baseline threshold is set to 144 days.

Considering the topographic complexity of the study area and the spatio-temporal resolution of SAR imagery, we applied strict baseline thresholds to minimize spatial and temporal decorrelation effects. The perpendicular baseline threshold was set to 315 m, and the temporal baseline threshold was set to 144 days [28,29]. The spatio temporal baseline map is shown in Figure 11. After baseline screening, 33 high-quality SAR images were retained for subsequent interferometric processing.

(2) Differential Interferogram Generation and Phase Unwrapping

Differential interferograms were generated by removing topographic phase contributions using an external DEM. Phase unwrapping was performed using the Minimum Cost Flow (MCF) algorithm [30], which is particularly suitable for regions with moderate to high coherence. The key unwrapping parameters were configured as follows:

Unwrapping step size: 1

Coherence threshold: 0.3

Intensity threshold: 0.02

(3) Atmospheric Phase Screen (APS) Correction

Atmospheric phase artifacts were mitigated using the adaptive filtering approach implemented in GAMMA software, which applies spatial and temporal filtering to separate atmospheric signals from deformation signals [31].

(4) Time-Series Inversion

The unwrapped differential phases were inverted using the Singular Value Decomposition (SVD) method to derive the time-series deformation and mean velocity maps [29].

**Figure 12:**
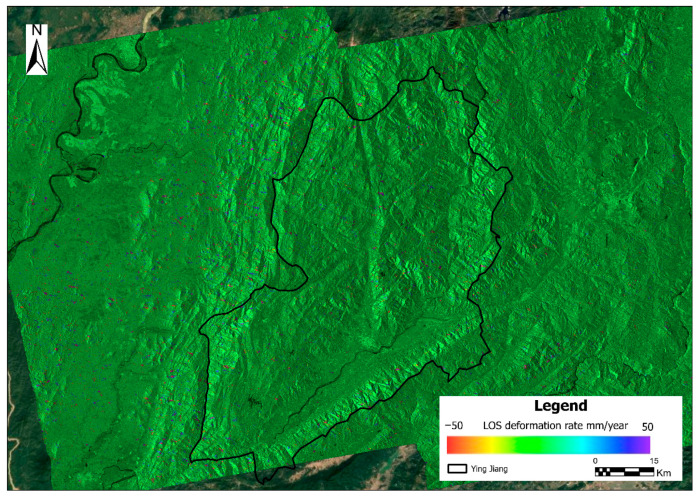
InSAR LOS deformation rate (mm/year). Only the InSAR data covering the study area are processed using the Gamma software.

All deformation data are processed on the laboratory server, and the software we use is Gamma software. The deformation rate map obtained in this study is shown in Figure 12.

The range of the ascending orbit is marked in the previous figure. However, in practice, only the InSAR data covering the study area are processed using the Gamma software.

### 4.4. Landslide Susceptibility Mapping for GWRF

**Figure 13:**
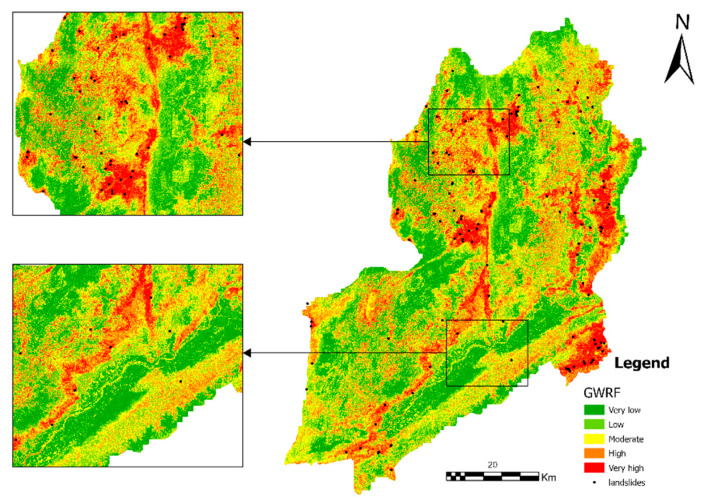
Landslide susceptibility mapping using the GWRF model. Two sub-regions are magnified to better illustrate the detailed susceptibility patterns and model performance in areas with different topographic characteristics.

**Figure 14:**
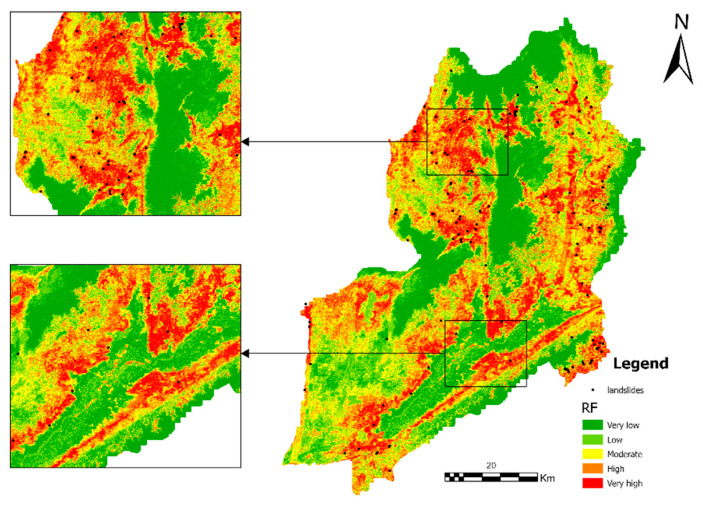
Landslide susceptibility mapping using the RF model. Two sub-regions are magnified to better illustrate the detailed susceptibility patterns and model performance in areas with different topographic characteristics.

**Figure 15:**
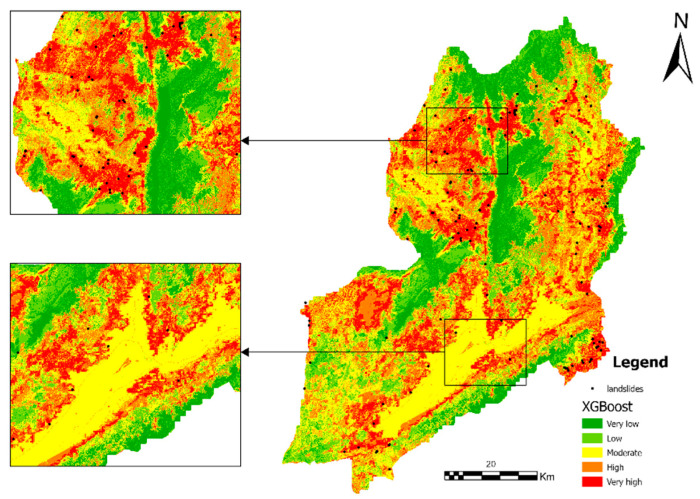
Landslide susceptibility mapping using the XGBoost model. Two sub-regions are magnified to better illustrate the detailed susceptibility patterns and model performance in areas with different topographic characteristics.

The landslide susceptibility zoning map generated based on the GWRF model is shown in Figure 13, while the corresponding results produced by the RF and XGBoost models are presented in Figure 14 and Figure 15, respectively.

The study area has been found to demonstrate significant spatial variability with regard to its landslide susceptibility. This susceptibility can be divided into five levels: extremely low, low, medium, high, and extremely high. Areas exhibiting either extremely low or low susceptibility are predominantly situated within regions characterised by relatively flat terrain and stable surface conditions. These regions collectively account for a substantial proportion of the study area. Medium susceptibility areas manifest in stripe or patch configurations, predominantly situated within the transition zones between disparate geomorphic units.

The local magnified map provides further evidence that landslide points are distributed in high concentrations within key areas, specifically in the high susceptibility zones. In contrast, the number of landslide points is comparatively low in the low susceptibility zones. This observation serves to verify the model’s discriminatory capability at the local scale. The susceptibility mapping results generated by the GWRF model exhibit clear spatial differentiation characteristics and high practical interpretability. In the ensuing text, a comparison is drawn between the susceptibility mapping of the GWRF model and that of the RF and XGBoost models.

In order to facilitate the interpretation of the landslide susceptibility map, the calculated Landslide Susceptibility Index (LSI) values were classified into five distinct categories: very low, low, moderate, high, and very high. The natural discontinuity optimization algorithm was adopted for this classification [32,33]. The selection of this method is predicated on its ability to reduce the variance within classes and maximise the variance between classes. This process enables the identification of groupings inherent in the data. In comparison to alternative classification schemes, such as Equal Interval or Standard Deviation, the Natural Breaks method is more effective in delineating the boundaries of hazard levels in non-normally distributed geological data. This ensures that high-susceptibility zones are accurately highlighted.

The proposed methodology is founded upon an algorithm that is designed to minimise the variance within classes while maximising the variance between classes. This ensures that distinct zoning is achieved. The specific probability thresholds determining the upper bounds of these classes were identified as 0.270, 0.396, 0.521, 0.666, and 1.000, respectively.

### 4.5. Evaluation of Model Performance

**Figure 16:**
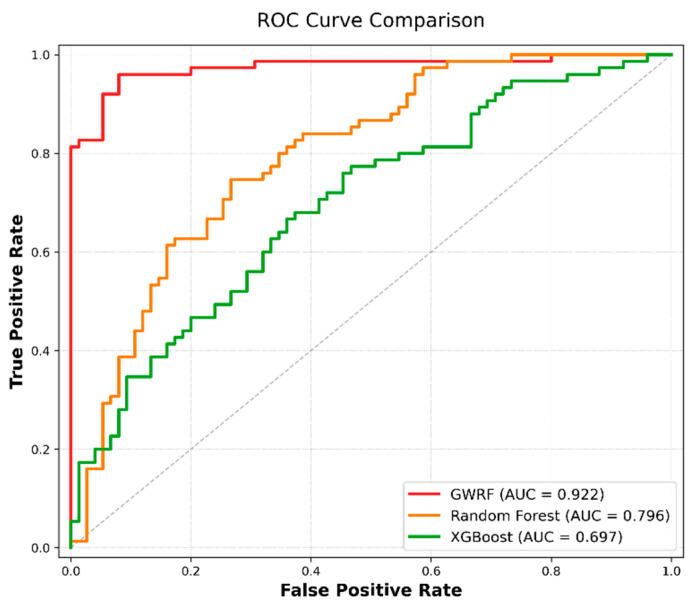
ROC curves of the three models.

Finally, the Receiver Operating Characteristic (ROC) curves were generated to visually evaluate the overall performance of the three models, as illustrated in Figure 16. The Area Under the Curve (AUC) values derived from the validation datasets are a pivotal indicator of the models’ predictive capabilities.

The findings indicate that the GWRF model demonstrated optimal performance, attaining a Validation AUC of 0.922. This finding suggests an excellent goodness-of-fit and a high probability of correctly ranking positive landslide samples. In comparison, the RF and XGBoost models exhibited relatively lower performance, with Validation AUC values of 0.796 and 0.697, respectively. The ROC analysis further confirms the superiority of the GWRF model in capturing the spatial heterogeneity of landslide susceptibility compared to the global regression models.

### 4.6. Model Performance Comparison

**Figure 17:**
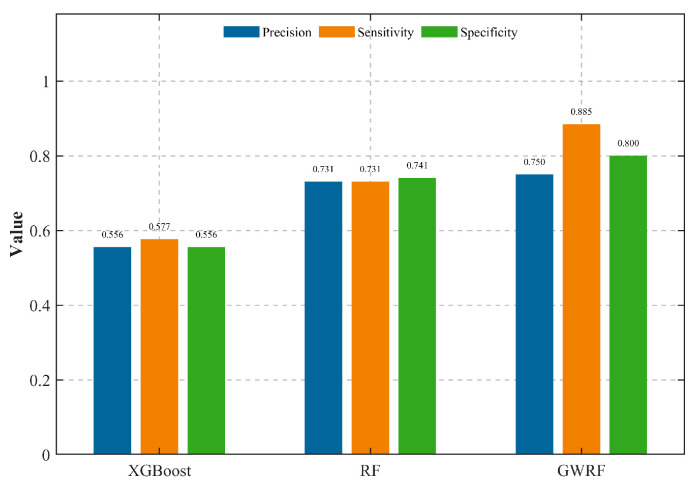
Performance metrics of three landslide susceptibility models. Blue bars represent Precision, orange bars represent Sensitivity, and green bars represent Specificity.

The objective of this study was to evaluate the predictive accuracy of the proposed models. In order to do this, the prediction results were compared against the 30% independent landslide testing dataset. As illustrated in Figure 17, the calculated values for precision, sensitivity, and specificity are presented.

The findings suggest that the GWRF model demonstrated superior performance in comparison to both the RF and XGBoost models across the three evaluation metrics. Specifically, the GWRF model demonstrated the highest level of sensitivity (0.885), thereby exhibiting superior capability in accurately identifying actual landslide occurrences in comparison to RF (0.731) and XGBoost (0.577). In addition, the GWRF model demonstrated the highest levels of precision (0.750) and specificity (0.800), thereby substantiating its efficacy in minimising false alarms. Conversely, the XGBoost model demonstrated the poorest performance, with all metrics falling below 0.60.

**Figure 18:**
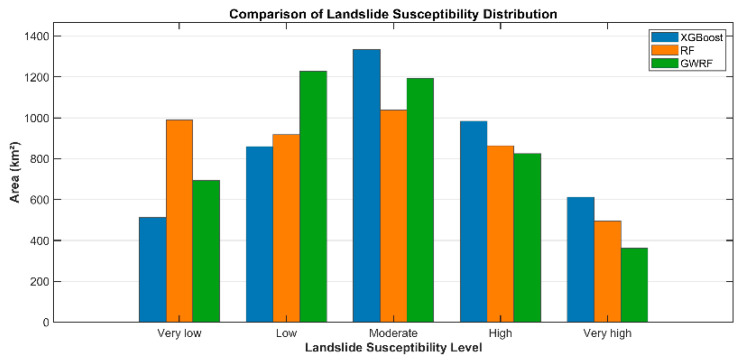
Statistics on landslide susceptibility area distribution for three models. Blue bars represent XGBoost, orange bars represent RF, and green bars represent GWRF.

The spatial distribution of susceptibility classes exhibited notable variation among the three models, particularly within the high-risk categories(Figure 18 and Table 3). The XGBoost model was able to successfully identify the most extensive hazardous areas, with 14.23% of the study area being classified as “Very High” susceptibility and 22.86% as “High” susceptibility. In comparison, the RF model demonstrated a moderate distribution, with “Very High” and “High” susceptibility areas accounting for 11.50% and 20.06%, respectively. The GWRF model yielded the most conservative estimates for high-risk zones; it classified only 8.42% of the area as “Very High” susceptibility and 19.18% as “High” susceptibility. The total area utilised for the calculation in all three models was found to be constant, measuring 4302.567 km^2^.

## 5. Discussion

### 5.1. Advantages of the GWRF Model

Recent studies have increasingly applied machine-learning techniques to construct reliable landslide susceptibility models; however, most of these approaches assume spatial stationarity and do not explicitly consider the spatial heterogeneity of landslide occurrence. Although some studies have introduced Geographically Weighted Regression (GWR) to address spatial heterogeneity, representing complex landslide-related geological processes using simple kernel functions remains challenging. In addition, when multiple landslide conditioning factors and large datasets are involved, global machine-learning models may produce biased susceptibility estimates.

To address these issues, this study adopts a GWRF model. The GWRF model exhibits two main advantages. (1), unlike traditional black-box machine-learning models, GWRF introduces spatial adaptability, allowing the relationships between landslide occurrence and conditioning factors to vary across space. This spatially adaptive framework enables local modeling of landslide susceptibility rather than enforcing a single global relationship. (2), compared with RF and XGBoost, GWRF demonstrates a stronger capability to capture spatial heterogeneity, thereby reducing the tendency of global models to overemphasize certain factors, such as distances to roads or rivers, which may lead to biased susceptibility estimations.

To further explore spatial differences among models, landslide susceptibility maps generated by GWRF were subtracted from those produced by RF and XGBoost. The resulting difference maps reveal pronounced spatial heterogeneity. In these maps, red and green pixels represent the magnitude and direction of the differences after subtraction rather than absolute susceptibility levels. Compared with RF, areas originally classified as high susceptibility tend to become more conservative under the GWRF framework, accompanied by noticeable changes in the spatial extent of high-susceptibility zones. Similar spatial heterogeneity is observed in the comparison between GWRF and XGBoost.

**Figure 19:**
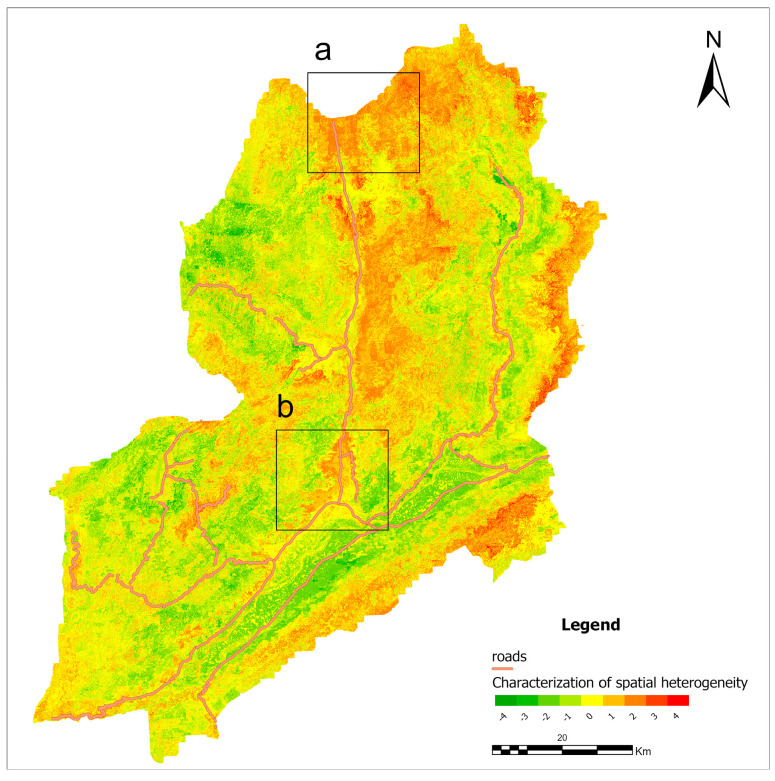
Difference map between GWRF and RF overlaid with road networks. Labels (a) and (b) indicate areas with intersecting roads.

As shown in Figure 19, discrepancies between GWRF and RF are mainly concentrated in regions a and b, where major transportation corridors are present. In these areas, slope instability is strongly influenced by engineering activities, including road construction and slope cutting. By calculating the Euclidean distance between landslide locations and roads, RF tends to assign higher susceptibility to areas within 1–2 km of road networks, which may result in an overestimation of landslide risk in road-intersection zones.

**Figure 20:**
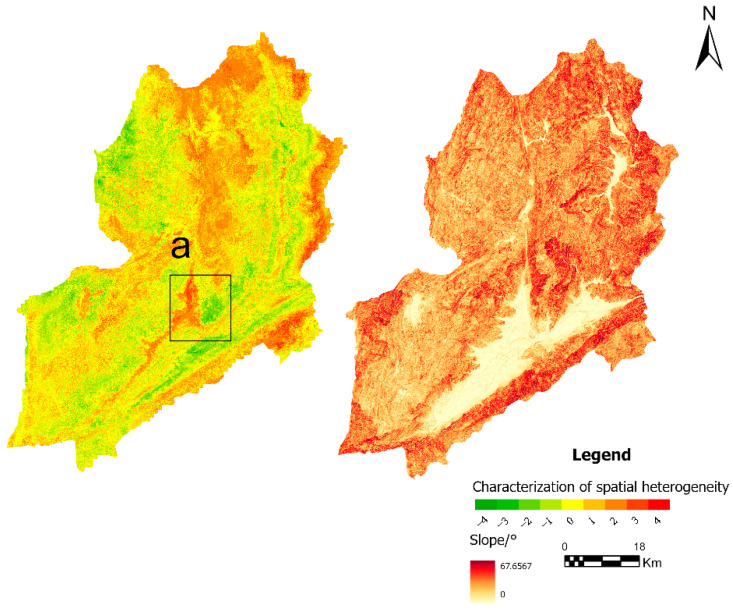
Difference map between GWRF and XGBoost with the slope map overlaid. Label (a) indicates an area with a gentle slope that was incorrectly identified as a high susceptibility zone by the XGBoost model.

In contrast, as shown in Figure 20, differences between GWRF and XGBoost are most evident in region a, characterized by strong terrain dissection and loose soil materials. Landslide occurrence in this area is strongly controlled by slope conditions. Analysis of the spatial relationship between landslide locations and slope indicates that XGBoost exhibits overfitting in this region and fails to adequately capture the spatial associations between terrain-related factors and landslide occurrence, leading to an overestimation of susceptibility.

### 5.2. Quantitative Validation Using InSAR Data

**Figure 21:**
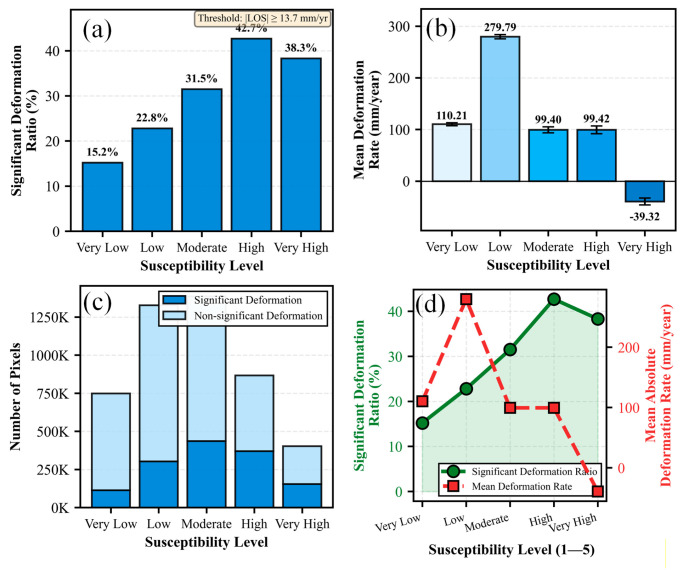
InSAR-based quantitative validation of susceptibility zonation. (**a**) Significant deformation ratio; (**b**) Mean deformation rate with standard deviation; (**c**) Pixel distribution by deformation significance; (**d**) Integrated trend analysis of deformation metrics versus susceptibility level.

A comprehensive quantitative statistical analysis was conducted on InSAR-derived deformation data encompassing 4,730,220 pixels across the study area to validate the accuracy of landslide susceptibility zonation [34]. The results reveal significant variations in deformation characteristics among different susceptibility classes [35,36] (Figure 21).

Regarding spatial distribution, the Moderate susceptibility zone contains the highest number of pixels (1,385,861 pixels, 29.3%), followed by the Low susceptibility zone (1,327,071 pixels, 28.1%) and Very Low zone (748,324 pixels, 15.8%). The High and Very High susceptibility zones comprise 866,500 pixels (18.3%) and 402,464 pixels (8.5%), respectively, exhibiting a decreasing trend with increasing susceptibility level.

In order to statistically assess the association between deformation characteristics and susceptibility zonation, active deformation pixels were defined as those exhibiting an absolute SBAS-derived LOS deformation rate exceeding 13.7 mm/yr. This threshold was rigorously determined based on the 3-sigma (3σ) criterion (approximately three times the standard deviation of the velocity field), representing a 99.7% confidence interval to distinguish significant ground movement from measurement noise. The proportion of active deformation pixels was subsequently calculated for each susceptibility class to reveal the gradient of instability across different hazard levels.

The comprehensive statistical analysis reveals a statistically significant association and a complementary relationship between InSAR-monitored surface deformation and landslide susceptibility zonation [36,37], rather than a simple linear consistency. While susceptibility assessment delineates long-term potential instability based on static conditioning factors, InSAR monitoring captures current surface dynamics.

Independent samples *t*-tests confirmed a significant difference in mean deformation rates between high-risk and low-risk zones (t = −20.49, *p* < 0.001). This distinction was further corroborated by the non-parametric Mann–Whitney U test (Z = −19.68, *p* < 0.001) and one-way ANOVA (F = 666.28, *p* < 0.001), as detailed in Table 4.

These results indicate that high susceptibility zones are statistically more likely to exhibit active deformation signatures (subsidence or downslope movement). The integration of these two independent datasets—one predictive (susceptibility) and one observational (InSAR)—provides mutually reinforcing insights, validating that high-susceptibility areas warrant priority attention for risk mitigation [38].

### 5.3. Relationship Between Lithology and Landslide Susceptibility Patterns

**Figure 22:**
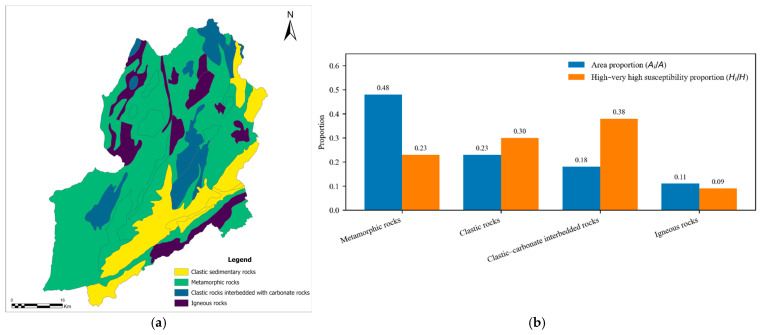
Relationship between Lithology and Landslide Susceptibility Patterns. (**a**) Lithology map; (**b**) The proportion of lithology *i* in the total area of the study region and the proportion of lithology *i* within the high–very high susceptibility zones.

To evaluate the geological rationality of the landslide susceptibility results, the susceptibility map classified by the natural breaks method was analyzed in conjunction with the lithological characteristics of the study area(Figure 22). Four major lithological types were identified: metamorphic rocks, clastic rocks, clastic rocks interbedded with carbonate rocks, and igneous rocks.

For each lithological type, the areal proportion ($A_{i}/A$) and its contribution to the high–very high susceptibility zones ($H_{i}/H$) were calculated and compared.

- ($A_{i}/A$): the proportion of lithology *i* in the total area of the study region.
- ($H_{i}/H$): the proportion of lithology *i* within the high–very high susceptibility zones (i.e., the lithological composition of the high-susceptibility zones).

The results indicate clear differences among lithologies. Metamorphic rocks occupy approximately 48% of the study area but account for only about 23% of the high–very high susceptibility zones, suggesting a relatively low susceptibility. In contrast, clastic rocks interbedded with carbonate rocks cover only about 18% of the total area, yet contribute nearly 38% of the high–very high susceptibility zones, indicating a pronounced enrichment in highly susceptible areas. Clastic rocks show moderate enrichment, while igneous rocks exhibit the lowest contribution to high-susceptibility zones.

### 5.4. Important Characteristics of LCFs

**Figure 23:**
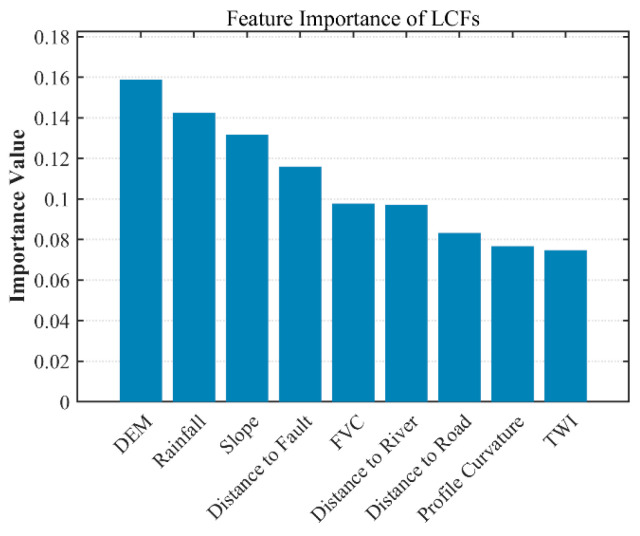
LCFs importance of GWRF model.

The feature importance results derived from the GWRF model are illustrated in Figure 23, which presents the outcomes of the feature importance analysis. The results indicate that the most influential landslide conditioning factors are DEM, precipitation, and slope gradient, as these variables contribute most significantly to landslide susceptibility. In particular, DEM and precipitation exhibit relatively high weights in the model, suggesting that they play a dominant role in controlling the spatial patterns of landslide occurrence. This finding is consistent with previous studies reported in the literature [39].

In contrast, other factors such as distance to road and TWI indices show relatively lower contributions. Although these factors may influence landslide occurrence in certain local areas, their overall impact on regional landslide susceptibility is limited. By quantitatively analyzing the importance of different conditioning factors, this study identifies the primary drivers of landslide susceptibility distribution, providing valuable references for future landslide risk prediction and disaster prevention and mitigation efforts [39,40].

### 5.5. Field Validation

**Figure 24:**
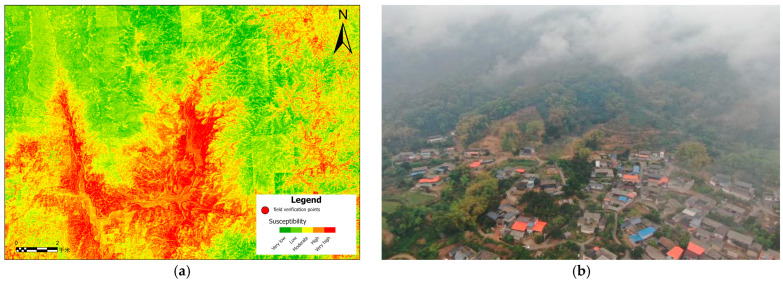

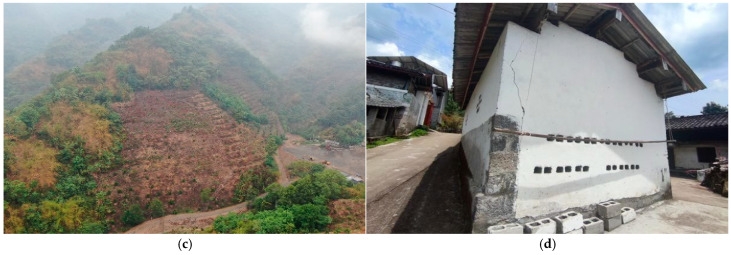
(**a**) Landslide susceptibility map generated using the GWRF model; (**b**) UAV aerial photograph of the corresponding area; (**c**) a confirmed landslide site identified through field investigation; and (**d**) field photograph showing deformation features of a residential building, indicating active slope instability in the high-susceptibility zone.

As demonstrated in Figure 24, Figure 24a presents the landslide susceptibility mapping results that have been generated based on the GWRF model, in conjunction with the location annotation of the field survey area. The field survey area is situated within the area of highest susceptibility, as identified by the model. Furthermore, the surrounding regions demonstrate a consistent distribution of high susceptibility characteristics. Figure 24b presents the drone aerial imagery of the area, which reveals significant slope undulations and the presence of houses and villages below. As illustrated in Figure 24c, the landslide phenomenon was observed on-site within the designated area. The landslide mass was clearly delineated, with distinct boundaries that defined its extent. Figure 24d presents a field photograph of cracks manifesting in buildings within the designated area. The field survey results demonstrate a high degree of spatial consistency with the GWRF susceptibility mapping, thereby providing further validation of the model’s reliability.

### 5.6. Landslide Susceptibility Map and Disaster Management

**Figure 25:**
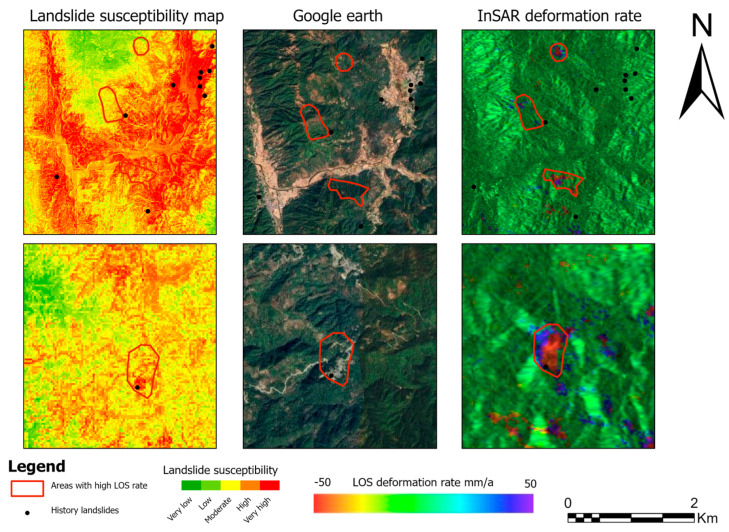
GWRF susceptibility, Google Earth imagery, and InSAR deformation rate.

The GWRF-based landslide susceptibility map provides a reliable spatial framework for identifying priority areas in landslide hazard prevention and mitigation. The model has identified high- and very-high-susceptibility zones, which can be used to guide targeted monitoring, particularly when integrated with InSAR-derived surface deformation data to detect potential slow-moving or incipient slope instabilities. Furthermore, high-resolution optical imagery from Google Earth offers complementary visual evidence of surface disturbances and infrastructure damage, thereby supporting rapid interpretation and field validation. The integration of susceptibility mapping, deformation monitoring, and optical image interpretation within this approach serves to enhance the efficiency and effectiveness of landslide risk management(Figure 25).

### 5.7. Limitations of the Innovative Method

It is important to note that although rainfall is treated as a background conditioning factor in this study, its inclusion may increase the sensitivity of the susceptibility results to hydro-climatic variability. This underscores the fundamental distinction between long-term landslide susceptibility assessment and short-term, trigger-based hazard prediction.

It is recommended that future research efforts focus on further delineating these two components by incorporating event-based rainfall data for dynamic hazard assessment, while maintaining background climatic indicators for susceptibility mapping.

A further limitation is evident in the composition of the landslide inventory. The historical landslide data utilised in this study does not differentiate between specific landslide types (e.g., shallow versus deep-seated landslides) or strictly define a minimum mappable size. Conversely, landslide locations were utilised as sample points for training machine learning models, with susceptibility evaluation conducted at a 30-m grid resolution. While this approach is effective in identifying general slope instability across the region, it is important to note that it aggregates different failure mechanisms that may respond differently to conditioning factors. For instance, deep-seated landslides are often controlled by geological structure and lithology, whereas shallow landslides are more sensitive to rainfall and vegetation cover. Future research could benefit from the classification of landslides by type in order to construct separate susceptibility models, thereby further refining the understanding of spatially heterogeneous controlling mechanisms.

### 5.8. Experimental Environment

In this study, the InSAR time-series processing was conducted on a workstation equipped with an Intel Xeon Gold 6534 CPU (8 cores, 16 threads, 3.9 GHz, Intel, Santa Clara, CA, USA) and an NVIDIA A2000 GPU (12 GB memory, NVIDIA, Santa Clara, CA, USA), requiring approximately 100 h to complete the deformation calculation. The model training and susceptibility mapping were performed on a separate workstation (Intel Core i7-14700KF CPU and NVIDIA GeForce RTX 4070 Ti SUPER GPU), with a total training time of approximately 2 h.

## 6. Conclusions

In this study, a Geographically Weighted Random Forest (GWRF) model was developed for landslide susceptibility mapping by integrating the advantages of Random Forest and Geographically Weighted Regression. The core methodological contribution of this work lies in embedding spatial weighting into a machine learning framework, enabling the model to explicitly account for spatial heterogeneity in the relationships between landslide occurrence and conditioning factors.

(1) Comparative analyses demonstrate that the GWRF model outperforms conventional RF and XGBoost models in terms of predictive accuracy and spatial discrimination ability. While traditional machine-learning models can be effectively applied to landslide susceptibility mapping, the results of this study indicate that models incorporating spatial heterogeneity provide more reliable and spatially consistent susceptibility patterns, particularly in geologically complex mountainous regions.
(2) The GWRF model exhibits distinct advantages in spatial adaptability compared to traditional black-box models. By allowing the relationships between landslide occurrence and conditioning factors to vary across space, the GWRF framework achieves local modeling of susceptibility rather than enforcing a single global relationship. This capability effectively captures spatial heterogeneity and mitigates the tendency of global models (such as RF and XGBoost) to overemphasize certain factors—such as distances to roads or rivers—thereby yielding more physically realistic and unbiased susceptibility estimations.
(3) Despite these promising results, several limitations should be acknowledged. Local residuals in the geographically weighted regression component may remain relatively large in some areas, and landslide inventory data are inevitably affected by investigation difficulty and limited field validation, as field surveys were conducted only in selected sub-regions. These factors may introduce uncertainty into the model outputs.
(4) Future work will focus on incorporating a broader range of landslide conditioning factors, improving the quality of InSAR deformation data, and integrating additional multi-source datasets to further refine and validate the GWRF framework. Overall, this study demonstrates that spatially aware machine-learning models represent a robust and promising direction for landslide susceptibility mapping and hazard assessment.

## Figures and Tables

**Table 1:** Main data source.

Data	Data Source	Data Description	Data Accuracy and Type
DEM	Geospatial Data Cloud site, Computer Network Information Center, Chinese Academy of Sciences (http://www.gscloud.cn) (accessed on 20 December 2025)	DEM data are derived from ASTER GDEM data.	30 m/Grid
Aspect	Derived from DEM data	Aspect data are derived from DEM data processing.	30 m/Grid
Slope	Derived from DEM data	Slope data are derived from DEM data processing.	30 m/Grid
Curvature	Derived from DEM data	Curvature data are derived from DEM data processing.	30 m/Grid
TWI	Derived from DEM data	TWI data are derived from DEM data processing.	30 m/Grid
FVC	Geospatial Data Cloud site, Computer Network Information Center, Chinese Academy of Sciences (https://www.gscloud.cn) (accessed on 20 December 2025)	FVC data are obtained using Landsat-8 Operational Land Imager (OLI) images.	30 m/Grid
Precipitation	National Tibetan Plateau Scientific Data Center (https://data.tpdc.ac.cn/) (accessed on 20 December 2025)	Precipitation data are derived from the National Tibetan Plateau Scientific Data Center.	1 km/Grid
Distance To River	OpenStreetMap (https://www.openstreetmap.org) (accessed on 20 December 2025)	Obtained through distance to the Nearest river.	30 m/Grid
Distance To Road	OpenStreetMap (https://www.openstreetmap.org) (accessed on 20 December 2025)	Obtained through distance to the Nearest road.	30 m/Grid
Distance To Fault	Yunnan geological survey (https://ynddj.org.cn/) (accessed on 20 December 2025)	Obtained through distance to the Nearest fault.	30 m/Grid
landslide inventory	Yunnan geological survey (https://ynddj.org.cn/) (accessed on 20 December 2025)	The landslide inventory was compiled based on field survey data obtained from 2021 to 2025, provided by Yunnan Geological Survey.	Specific longitude and latitude information/Point

**Table 2:** Radar parameter.

Data Type	Sentinel-1A
Frame Start-Frame End	172–1262
Revisit Cycle	12 day
Polarization Mode	VV
Acquisition Mode	IW
Spatial Resolution	5 m × 20 m
number of images	33
Satellite flight mode	Ascending

**Table 3:** Area and percentage of each susceptibility class in the study area.

Model	Landslide Susceptibility	Area Ratio (%)	Area (km^2^)
XGBoost	Very low	11.93%	513.144
	low	19.96%	858.843
	Moderate	31.02%	1334.493
	High	22.86%	983.7756
	Very high	14.23%	612.3114
RF	Very low	22.99%	989.2827
	low	21.32%	917.2503
	Moderate	24.13%	1038.3165
	High	20.06%	862.9317
	Very high	11.50%	494.7858
GWRF	Very low	16.11%	692.9883
	low	28.55%	1228.4397
	Moderate	27.74%	1193.7024
	High	19.18%	825.219
	Very high	8.42%	362.2176

**Table 4:** Results of Statistical Tests.

Test Method	Statistic	Degrees of Freedom	*p*-Value
Independent Samples *t*-test	t = −20.49	4,684,529	*p* < 0.001 ***
Mann–Whitney U Test	Z = −19.68	-	*p* < 0.001 ***
ANOVA	F = 666.28	-	*p* < 0.001 ***

Note: *** denotes a highly significant difference at the *p* < 0.001 level.

## Data Availability

The data presented in this study are available on request from the corresponding author. The data are not publicly available due to privacy restrictions.

## Abbreviations

The following abbreviations are used in this manuscript:

LSM	Landslide susceptibility mapping
GWRF	Geographically Weighted Random Forest
GWR	Geographically Weighted Regression
RF	Random Forest
LCF	Landslide Conditioning Factors
DEM	Digital Elevation Model
LOS	Line-Of-Sight
XGBoost	eXtreme Gradient Boosting
InSAR	Interferometric Synthetic Aperture Radar
TWI	Topographic Wetness Index
